# Development of an Affimer-antibody combined immunological diagnosis kit for glypican-3

**DOI:** 10.1038/s41598-017-10083-w

**Published:** 2017-08-29

**Authors:** Chunmei Xie, Christian Tiede, Xuanyi Zhang, Congrong Wang, Zhixiong Li, Xiao Xu, Michael J. McPherson, Darren C. Tomlinson, Weiwen Xu

**Affiliations:** 1 State Key Laboratory of Organ Failure Research, Institute of Antibody Engineering, School of Laboratory Medicine and Biotechnology, Southern Medical University, Guangzhou, 510515 China; 2 BioScreening Technology Group, School of Molecular and Cellular, Biology, Faculty of Biological Sciences, University of Leeds, Leeds, LS2 9JT UK; 3 Astbury Centre for Structural Molecular Biology, Faculty of Biological Sciences, University of Leeds, Leeds, LS2 9JT UK; 4 First Affiliated Hospital of Zhejiang University, Hangzhou, 310003 China; 5 Department of Laboratory Medicine, Nanfang Hospital, Southern Medical University, Guangzhou, 510515 China; 6 R&D center, DaRui Biotechnology Co., Ltd, Guangzhou, 510655 China

## Abstract

Glypican-3 (GPC3) is a promising new marker for hepatocellular carcinoma, but the reported values for serum GPC3 differ markedly between currently available kits. Here we isolated Affimer non-antibody binding proteins against GPC3 by phage display and developed a new sandwich chemiluminescence immunoassay (CLIA) combining an Affimer with a monoclonal antibody (Affimer-MAb CLIA). The proposed CLIA assay demonstrated a wide linear range  0.03–600 ng/mL) with a good linear correlation coefficient (0.9999), a high detection limitation (0.03 ng/mL) and specificity (0–0.002%) for detection of GPC3. The accuracy, hook effect and stability were demonstrated to be satisfactory. The mean level of GPC3 in serum was higher (>8.5 fold, P < 0.001) in hepatocellular carcinoma patients compared to healthy and other liver disease individuals. A poor correlation (correlation coefficients ranged from −0.286 to 0.478) was observed through pairwise comparison within different kits. However, only this newly developed CLIA test showed high specificity and correlated with the “gold standard” GPC3-immunohistochemistry. This study indicates that Affimer-MAb CLIA can be used to generate a sensitive immunodiagnostic kit, which offers the potential for a highly specific clinically-relevant detection system.

## Introduction

Glypican-3 (GPC3) is a heparin sulfate proteoglycan molecule first identified by Pila and associates in patients with Simpson-Golabi-Behmel Syndrome in 1996^1^. Recent studies^2–5^ show that GPC3 is associated with the development and presence of hepatocellular carcinoma (HCC), and might serve as an auxiliary diagnostic marker for the disease. As a result, a GPC3 immunohistochemistry (IHC) kit has been developed and approved by China Food and Drug Administration (CFDA) for use in diagnosing HCC^6^. Moreover, numerous studies^7–10^ have detected GPC3 in the peripheral blood of HCC patients, and have reported that its concentration in serum indirectly reflects the number of GPC3 positive cells present *in* *vivo* ^11^. It has been reported that GPC3 is a serological marker, with the highest expression level of those markers currently found in alpha fetal protein (AFP) negative HCC^12^. The capability to detect GPC3 glycoprotein could assist in the early diagnosis of HCC and help to establish a clinical prognosis for HCC patients. While there is support for the diagnostic value of detecting GPC3 in serum^10, 13^, the current GPC3 detection kits remain of value only for research purposes rather than of clinical value.

Affimers are non-antibody binding proteins that constrain two 9-aa variable regions for molecular recognition. Affimers can mimic many important functions of antibodies, are highly stable and can be produced relatively cheaply, reproducibly and in high quantities using *E*. *coli* recombinant protein expression systems. Large phage display libraries have been generated^14^ and screened against hundreds of target proteins, peptides and small molecules to isolate Affimers for different applications, including diagnostics^15–18^. It has previously been shown that Affimer reagents can be isolated that are specific for a protein target and can discriminate between even highly related targets^14, 19^. Thus they have potential as an important alternative to antibodies for target molecule capture in diagnostic applications. The ability to use Affimers in sandwich ELISA’s has not yet been fully assessed and combining the use of Affimers with monoclonal antibodies in diagnostic kits may represent an important approach for altering their linear range, sensitivity and specificity.

Here, we report the development of a new sandwich chemiluminescence immunoassay (CLIA) that is much more sensitive than ELISA and can be undertaken on an automatic system based on a combination of an Affimer and a monoclonal antibody (MAb) for measuring GPC3 in serum (Affimer-MAb CLIA). The performance of the new proposed Affimer-MAb CLIA was assessed both with recombinant GPC3 protein and with clinical samples.

## Results

### Screening and generation of Affimers against GPC3

Phage display library screening was performed against immobilized GPC3 protein. Thirty two Affimer clones from the third panning round were tested for their ability to bind GPC3 by phage ELISA. Twenty nine showed binding to the target (see Supplementary Fig. S1) and DNA sequencing revealed eleven unique Affimers. Clones representative of the six most frequently identified sequences were initially assessed by CLIA for their ability to capture or detect GPC3.

### Screening of the paired core materials for CLIA

Six Affimers (GPC3-1, GPC3-4, GPC3-6, GPC3-21, GPC3-22, and GPC3-25) and 2 MAbs (8G6 and 7D11, prepared in our previous work^13^) were assessed for their ability to be used as reagents. The signal to noise ratio (SNR) was calculated using the formula: SNR = (luminescence value of 300 ng/mL standard)/(luminescence value of 0 ng/mL standard). The most efficient capture reagent was Affimer-GPC3-22, which showed the highest SNR (see Supplementary Table S1) when used in combination with all the other Affimers and MAb reagents. In fact the highest SNR was observed when Affimer-GPC3-22 was used in combination with MAb-7D11. Thus, Affimer-GPC3-22 as capture reagent and MAb-7D11 as detection reagent were prepared for the subsequent experiments for CLIA kit development.

### Development of the CLIA

**Figure 1:**
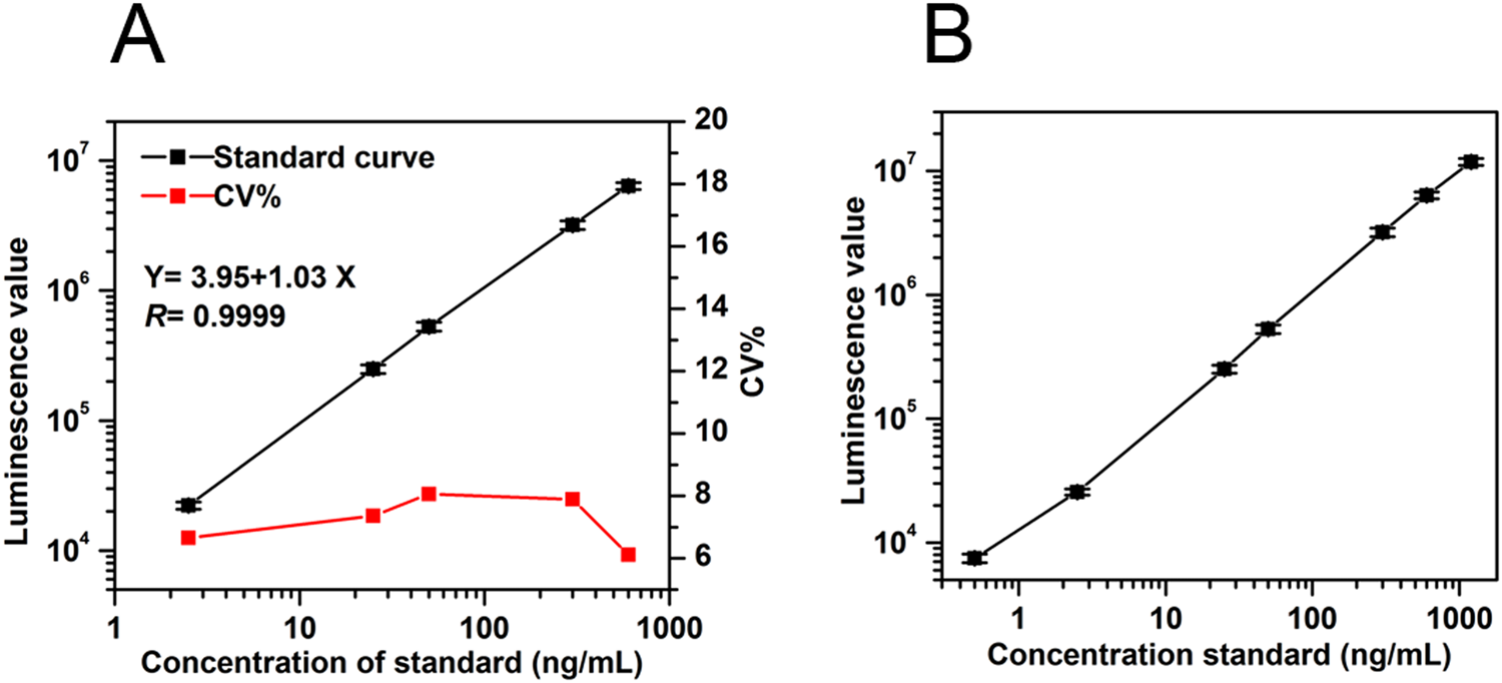
Standard curve and hook effect for the proposed Affimer-MAb CLIA assay (n = 3). (**A**) Standard curve for GPC3 and the intra-assay CV% for each concentration based on three independent replicates. (**B**) Hook effect for the proposed Affimer-MAb CLIA assay.

Using the Affimer as a capture reagent and the MAb for detection, a CLIA was developed (see Supplementary Fig. S2 for the schematic illustration). All the key parameters were optimized and six reference concentration points were set at 0, 2.5, 25, 50, 300 and 600 ng/mL. Through optimization, the results were as follows: The proportion of Affimer-GPC3-22 to magnetic beads was 1:80 (w/w). The ratio of MAb to NHS-LC-Biotin was 1:10 (w/w). The volume of acridinium ester (AE)-labeled streptavidin (SA) mixture used was 100 μl. The pH of the dilution buffer was 7.8. Under these optimized conditions a calibration curve for the proposed CLIA was developed (Fig. 1). The working curve equation (Fig. 1A) was Y = 3.95 + 1.03X with the linear correlation coefficient of 0.9999, and the coefficient of variation (CV) < 10%(n = 3).

### Analytic performance of the proposed CLIA assay

Initially the sensitivity and linear range were assessed. The lower limit of detection (LLD) of the assay was 0.03 ng/mL when the luminescence value (M + 2 SD) of 0 ng/mL standard (n = 20) was substituted into a standard curve equation. A biologically relevant concentration range from 0 to 1200 ng/mL was used to evaluate the linear range of the CLIA. The results show a wide linear range from 0.03 to 600 ng/mL and no hook effect was observed up to 1,200 ng/mL of GPC3 (Fig. 1B).

To evaluate cross-reactivity, several proteins known to be upregulated in HCC were selected, including alpha fetoprotein (AFP), carcinoembryonic antigen (CEA), carcino embryonic antigen19-9 (CA19-9), hepatitis B surface antigen (HBsAg) and Hepatitis C virus–core antigen (HCV-cAg). The cross-reactivity was calculated using the formula: cross-reactivity (%) = (measured concentration of GPC3)/(actual concentration of interferent). The cross-reactivity against these protein was low (0% to 0.002%) and had negligible effect on the simultaneous assay for GPC3 in human serum, which indicates our proposed assay is highly selective for GPC3.

The recovery rate was examined to evaluate the accuracy of the proposed assay. High concentrations of GPC3 (50 and 300 ng/mL) were added to existing samples containing GPC3 at concentrations of 0.5, 1 and 2 ng/mL, respectively. The mixed samples were retested for their GPC3 concentration. The recovery rates were within the range 91.88–104.50% (see Supplementary Table S2), which are in the acceptable range for accuracy of an immunoassay.

The precision of the proposed CLIA assay was also determined by examining its coefficient of variation (CV). Three concentrations (1, 5 and 50 ng/mL) were assayed twenty times in one day to assess the intra-assay precision, and on three sequential days with ten replicates to determine the inter-assay precision. The intra-assay CV and inter-assay CV were calculated (Table 1) and varied between 6.06% and 8.98%.

**Table 1:** Precision of the proposed assay.

Sample concentration (ng/mL)	Intra-assay precision (n = 20)		Inter-assay precision (n = 10 * 3)	
	Mean ± SD (ng/mL)	CV (%)	Mean ± SD (ng/mL)	CV (%)
1	0.92 ± 0.07	7.60	0.89 ± 0.08	8.98
5	4.29 ± 0.26	6.06	5.01 ± 0.40	7.98
50	44.07 ± 2.80	6.35	46.89 ± 3.35	7.14

SD: standard deviation; CV: coefficient of variation.

CV = (SD/Mean) ×100%.

To determine the stability of the proposed kit, sealed kits were stored at 2–8 °C for 0, 3, 6, and 12 months, and uncovered kits were stored at 2–8 °C for 0, 14, and 20 days. At each time point, the physical appearance and the analytical performance were evaluated to assess their stability. All of the stored kits, with the exception of the kit stored uncovered for 20 days, showed a satisfactory outcome. Therefore, the kits were stable for no less than 12 months, and the remaining reagents should be discarded 14 days after first use.

A comparison of analytical performance of the proposed assay with the dual-MAbs CLIA kit from DaRui was conducted. The results showed the proposed Affimer-MAb CLIA assay displayed a better detection limit, higher specificity, and a wider linear range (Table 2).

**Table 2:** The results of analytic performance within dual-MAbs CLIA kit and the proposed assay.

		dual-MAbs CLIA kit	Proposed assay
Detect limitation		0.05 ng/mL	0.03 ng/mL
Linearity		0.9999	0.99999
Linear range		0–500 ng/mL	0.03–600 ng/mL
Specificity		0.015–0.021%	0–0.002%
Accuracy		94.50–106.80%	91.80–104.53%
Precision	Intra-assay	4.76–5.42%	6.06–7.60%
	Inter-assay	6.41%	7.14–8.98%
Stability (2–8 °C)	sealed	12 months	12 months
	uncovered	30 days	14 days

CLIA: chemiluminescence immunoassay.

### Clinical detection performance of the proposed CLIA assay

**Figure 2:**
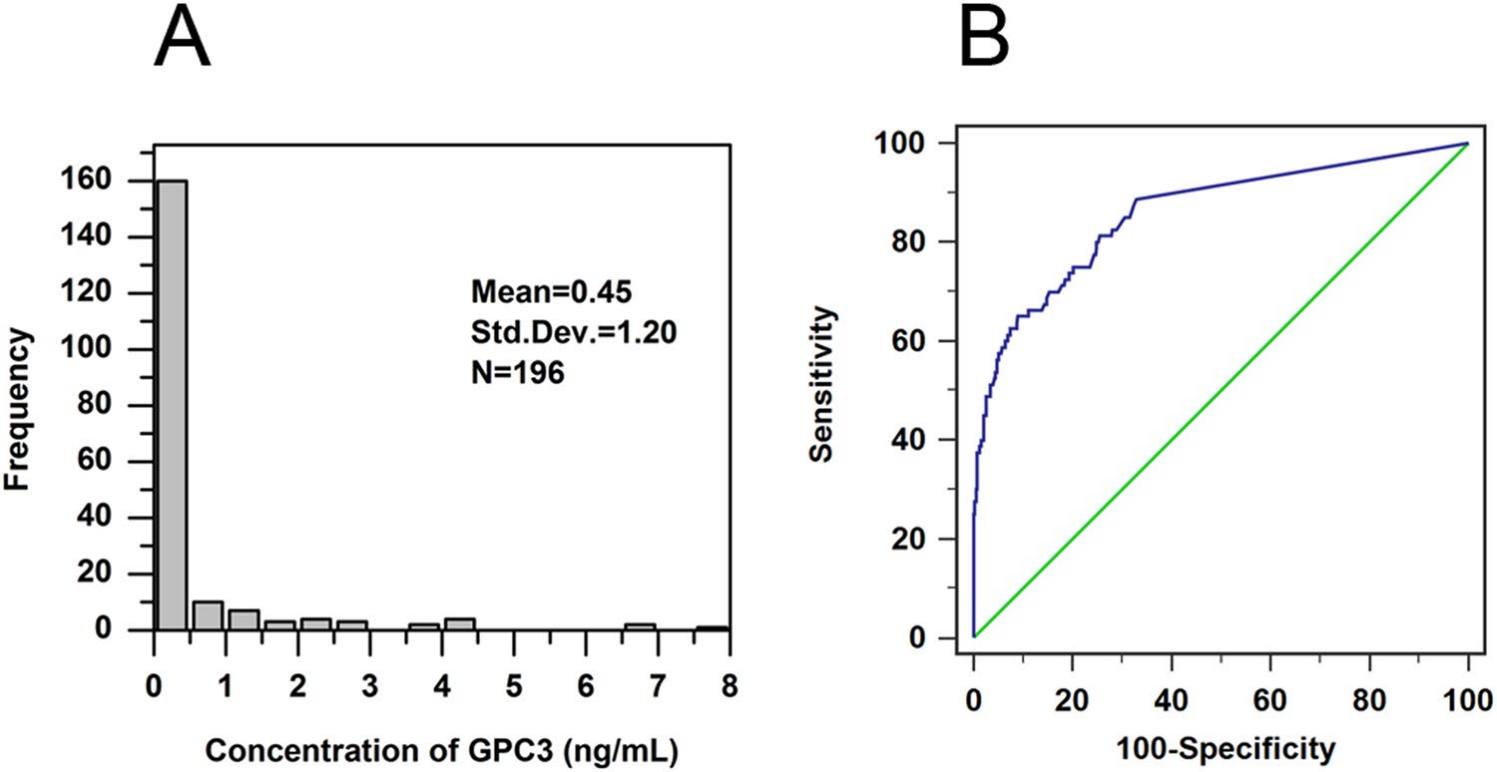
Cutoff value for the proposed Affimer-MAb CLIA assay. (**A**) Frequency distribution of GPC3 concentrations in a healthy population (n = 196). (**B**) The ROC curve of the proposed Affimer-MAb CLIA assay (n = 276). Green line represents the diagnostic reference line; blue line represents the ROC curve of GPC3.

To establish the cutoff value and normal range of GPC3 for the new Affimer-MAb CLIA kit in human serum, a total of 276 clinical serum samples were measured. Based on the frequency distribution data obtained from 196 healthy individuals (Fig. 2A), the cutoff value for GPC3 was initially set at 2.03 ng/mL (95% confidence interval). According to the ROC curve constructed based on the results from 80 patients with HCC and 196 healthy control subjects, the area under the ROC was 0.856 (Fig. 2B). The kit had a sensitivity of 65% and a specificity of 90.7% when the cut-off value was set at 0.925 ng/L. Based on comprehensive referencing to the frequency distribution, ROC curve and other reports, we finally set the cutoff value at 1.1 ng/mL, with a reasonable sensitivity of 62.5% (50/80) and specificity of 92.3% (181/196).

**Figure 3:**
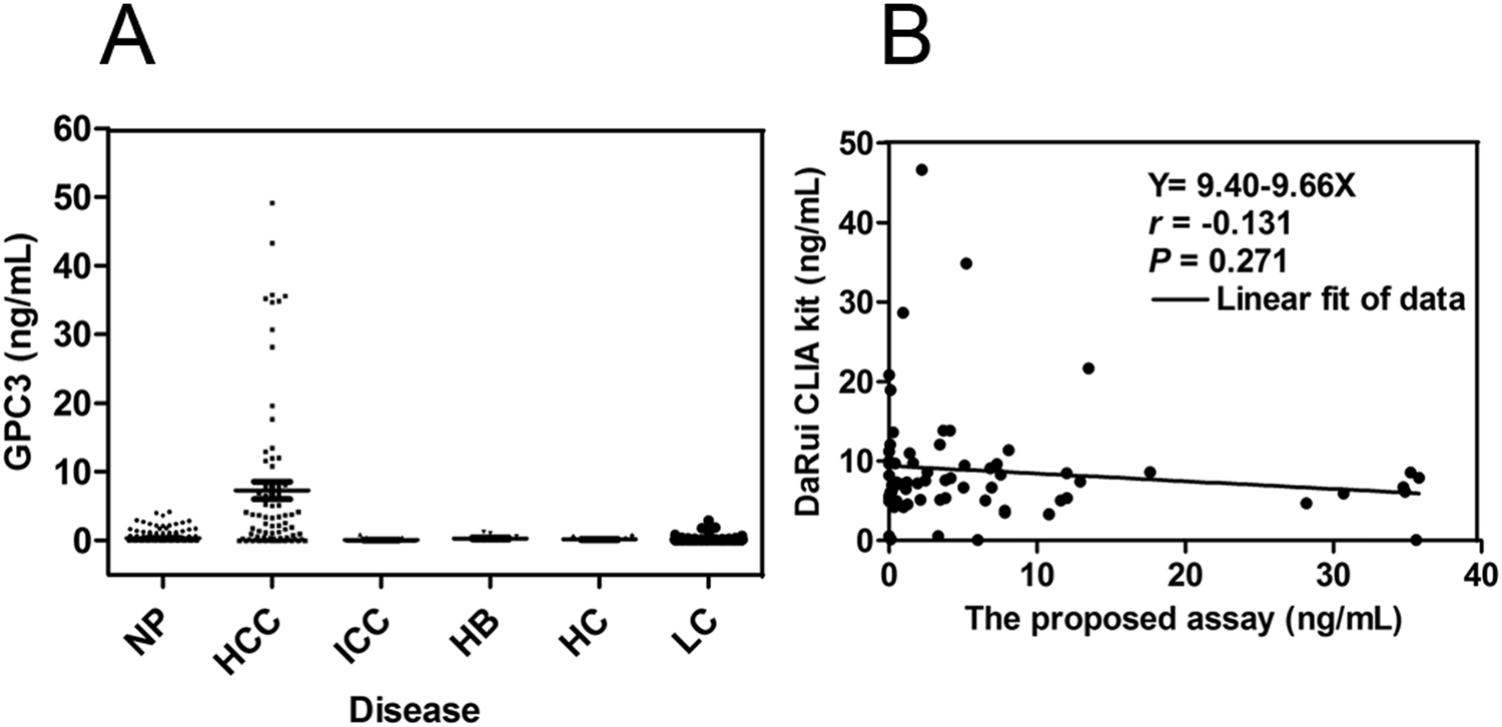
Quantitative detection results of GPC3 in clinical samples. (**A**) GPC3 protein in serum from 80 HCC patients and other patients with liver diseases or other cancers measured using the new developed kit. (NP: normal population; HCC: hepatocellular carcinoma; ICC: intrahepatic cholangiocarcinoma; HB: hepatitis B; HC: hepatitis C; LC: liver cirrhosis). (**B**) Comparison results of the GPC3 concentration in 73 serum from HCC patients between the proposed new assay and dual-MAbs CLIA from DaRui (DaRui CLIA).

Three hundred and seventy-five samples including 80 patients with HCC, 196 healthy individuals, 10 with hepatitis C, 10 with hepatitis B, 29 with intrahepatic cholangiocarcinoma and 50 patients with liver cirrhosis were evaluated using the Affimer-MAb CLIA assay. The GPC3 levels in different groups are shown in Fig. 3A. Serum GPC3 level was significantly higher in HCC (7.30 ± 11.42 ng/mL) than in healthy control (0.45 ± 1.20 ng/mL, *P* < 0.001), hepatitis C (0.16 ± 0.27 ng/mL, *P* < 0.001), hepatitis B (0.30 ± 0.47 ng/mL, *P* < 0.001), liver cirrhosis (0.28 ± 0.57 ng/mL, *P* < 0.001) and intrahepatic cholangiocarcinoma (0.07 ± 0.19 ng/mL, *P* < 0.001). Therefore, the proposed assay can appropriately differentiate HCC from healthy subjects and patients with other liver diseases.

**Figure 4:**
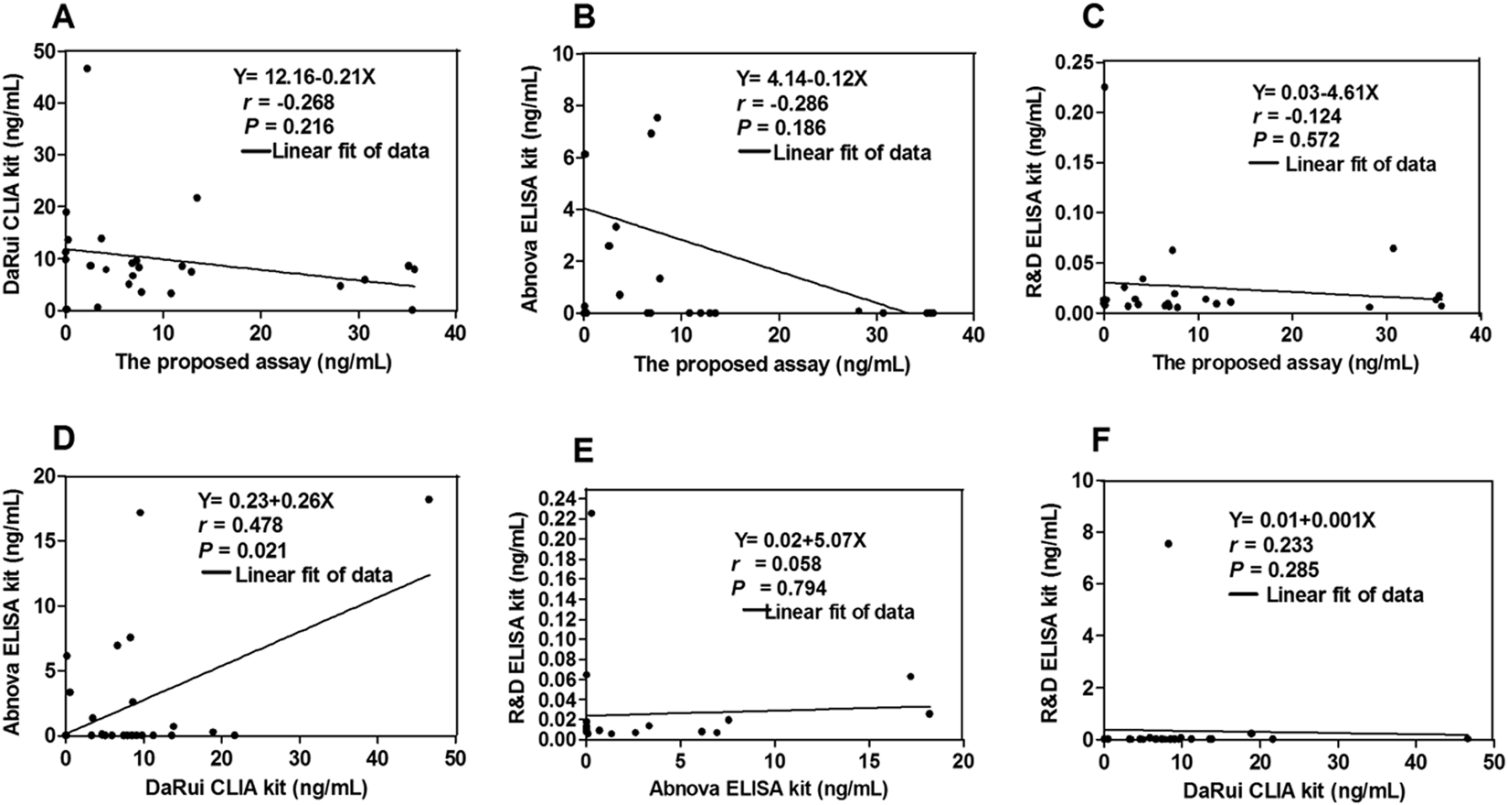
Comparison results between the four different kits. (**A**) Comparison between the proposed assay to dual-MAbs CLIA from DaRui(DaRui CLIA) (n = 25). (**B**) Comparison between the proposed assay to Abnova ELISA kit (n = 25). (**C**) Comparison between the proposed assay to R&D ELISA kit (n = 25). (**D**) Comparison of the DaRui CLIA kit to Abnova ELISA kit (n = 25). (**E**) Comparison of the Abnova ELISA kit to the R&D ELISA kit (n = 25). (**F**) Comparison of the DaRui CLIA kit to the R&D ELISA kit (n = 25).

GPC3 levels in 73 HCC serum samples were simultaneously detected by the proposed Affimer-MAb CLIA assay and the dual-MAbs CLIA kit from DaRui. The correlation was poor (Y = 9.40 + 9.66X, r = −0.1307, *P* = 0.2705 > 0.05, see Fig. 3B). Furthermore, GPC3 levels in 25 HCC serum samples were detected by the proposed Affimer-MAb CLIA assay and three other commercially available dual-MAbs kits (DaRui CLIA kit, Abnova ELISA kit, and R&D ELISA kit). The correlations between them are shown in Fig. 4. Poor correlation was obtained between the proposed Affimer-MAb CLIA assay and the kits from DaRui (r = −0.268, *P* = 0.216 > 0.05, see Fig. 4A), Abnova (r = −0.286, *P* = 0.186 > 0.05, see Fig. 4B) and R&D (r = −0.124, *P* = 0.572 > 0.05, see Fig. 4C). Meanwhile, the correlation between the kits from DaRui and Abnova was a little higher (r = 0.478, *P* = 0.021 < 0.05, see Fig. 4D).

Due to the lack of correlation between assay kits, we thus evaluated the correlation between the two CLIA kits and the gold standard immunohistochemistry test for diagnosing HCC using GPC3. Forty one pre-operative serum samples with paired tissue samples from HCC patients were assessed by the proposed Affimer-MAb CLIA assay and the dual-MAbs CLIA kit from DaRui, as well as by the IHC kit from LBP. Sensitivity and specificity between the kits were obtained by the McNemar’s test for correlated proportions. GPC3 was positively expressed in 78.05% (32/41) of the HCC tissues but none of the para-carcinoma tissues. Taking the IHC as the “gold standard control”, as shown in Table 3, the proposed Affimer-MAb CLIA assay had a sensitivity of 81.25% (26/32), specificity of 100% (9/9), and total coincidence of 85.36%, Kappa value 0.655. The dual-MAbs CLIA kit had a sensitivity of 90.62% (29/32) and a specificity of 0% (0/9), and total coincidence of 70.73%, Kappa value −0.123. The specificity and accuracy of the proposed assay is therefore more comparable to that of the IHC when used to diagnosis of HCC.

**Table 3:** Results of McNemar’s test comparing the proposed new assay and dual-MAbs CLIA kit to the IHC (n = 41).

		Proposed assay		Total	dual-MAbs CLIA kit		Total
		+	−		+	−	
GPC3- IHC	+	26	6	**32**	29	3	**32**
	−	0	9	**9**	9	0	**9**
Total		**26**	**15**	**41**	**38**	**3**	**41**

IHC: immunohistochemistry. +positive, −negative.

### Correlation between serum AFP and GPC3

**Figure 5:**
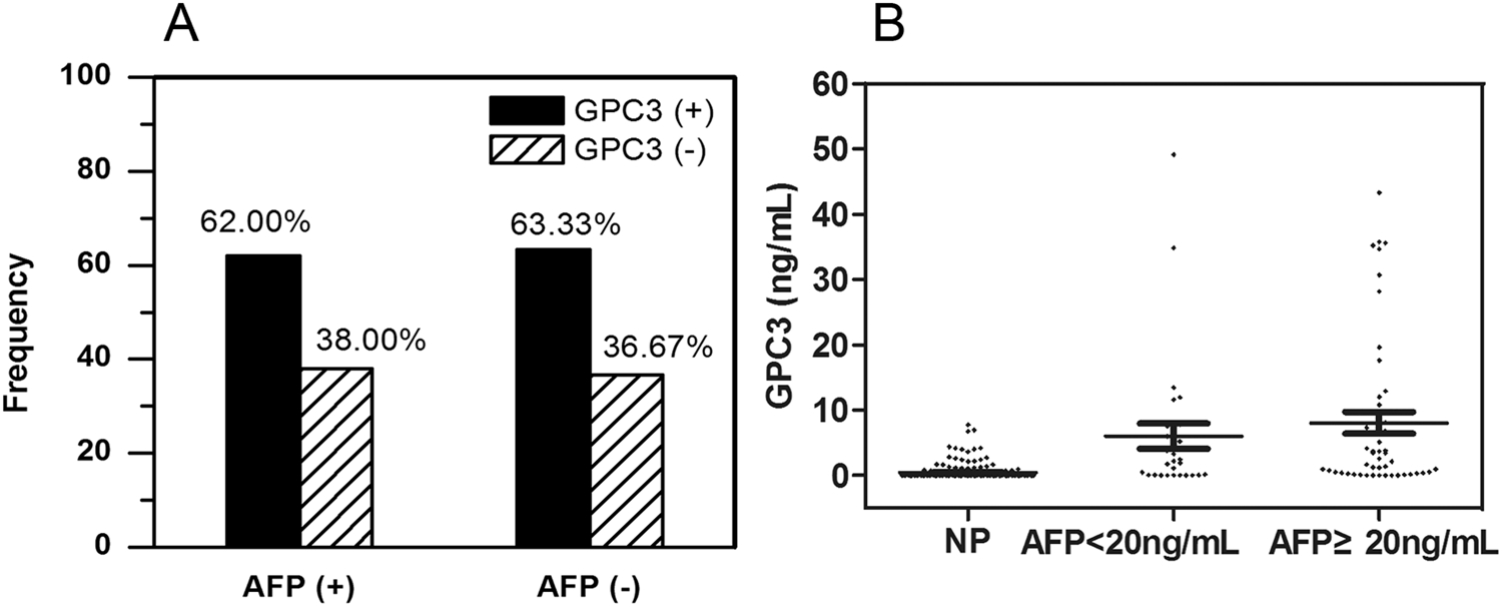
The Correlation between serum AFP and GPC3. (**A**) GPC3 positive rate in AFP positive group (n = 50) and AFP negative group (n = 30) in HCC patients. (**B**) GPC3 level in AFP < 20 ng/mL group (n = 30), AFP ≥ 20 ng/mL group (n = 50) in HCC patients and in 196 normal people (NP).

The serum AFP levels were also measured in 179 patients’ samples and 325 healthy volunteers. Serum AFP level was significantly higher in HCC (6105.64 ± 18271.30 ng/mL) than that in liver cirrhosis (1324.62 ± 7535.15 ng/mL, *P* < 0.001), healthy control (75.35 ± 922.15 ng/mL, *P* < 0.001), hepatitis B and hepatitis C (10.84 ± 29.17 ng/mL, *P* < 0.001), and intrahepatic cholangiocarcinoma (10.70 ± 20.99 ng/mL, *P* < 0.001) (see Supplementary Table S3). As shown in Fig. 5A, there was no difference within the positive rate of GPC3 in AFP positive group and AFP negative group in 80 HCC patients (62% *vs*. 63.33%, P > 0.05). There was no difference in the GPC3 level for the AFP < 20 ng/mL group and the AFP ≥ 20 ng/mL group in 80 HCC patients (6.04 ± 10.68 ng/mL *vs*. 8.05 ± 11.89 ng/mL, P = 0.47) (Fig. 5B). Thus the combination of AFP and GPC3 could increase the sensitivity to 87.50% for HCC diagnosis while it was only 57.50% and 62.50% for AFP or GPC3, respectively (see Supplementary Table S4).

## Discussion

GPC3 is a promising new tumor marker for HCC, and its clinical value for the diagnosis of HCC has been assessed and confirmed by a range of experts^4, 5, 20–24^. GPC3 was found to be highly expressed in HCC patients’ tissue and serum but not in CCA or normal liver tissue or serum. GPC3 may have a better utility value in detecting small and early HCC than alpha fetal protein. Furthermore, GPC3 is also a promising candidate for HCC therapy and the evaluation of GPC3 both in histology and serum are valuable companion diagnostics in GPC3-targeting therapy^25^. Whilst one histological kit has been approved by CFDA, there is no reliable serum kit. Although several serum detection assays^10, 26, 27^ have been reported, the sensitivity and working curves were variable. In addition, meta-analysis of the literature^28–30^ has suggested that GPC3 levels in serum are indeed higher in HCC than in normal subjects. However, the diagnostic utility has been questionable because the reported values differ considerably between the available assays. This is presumably due to the different antibodies used in different assays and the lack of an antibody with comprehensive scientific validation^31^. New alternatives to antibodies for immuno-diagnostic kit development are worth exploring to provide improved detection performance through increased target specificity. Non-antibody proteins selected from phage display libraries may provide such useful alternative detection reagents^32^.

In this study, we screened a non-antibody binding protein library and identified Affimer reagents specific for GPC3 for use in developing a sandwich chemiluminescence immunoassay (Affimer-MAb CLIA) for detection of GPC3 in serum. In comparison with the dual-MAbs CLIA kit from DaRui, that is a traditional immunoassay using two monoclonal antibodies (8G6 and 7D11)^10^, the only difference from the proposed Affimer-MAb CLIA assay was the capture reagent. In the CLIA assay developed here we introduced Affimer GPC3-22 as the capture reagent, rather than MAb 8G6 used in the dual-MAbs CLIA kit. The analytical performance of the proposed Affimer-MAb CLIA assay was better than that of the dual-MAbs CLIA, with a lower detection limit, higher specificity, and a wider linear range. Unlike the other kits tested, the proposed assay also differentiated HCC from other liver diseases (ICC, LC, HB, HC) and healthy control (>8.5 fold, P < 0.001). This difference is most likely due to better target specificity of the Affimer compared with the antibody reagents. Otherwise, the reported sensitivity of GPC3 in HCC diagnosis varied from 36% to 65%, and specificity from 55% to 100%, with substantial heterogeneity^33^. The CLIA test reported here does not represent a novel discovery, it represents an adaptation and improvement to a well-established clinical assay test format by the inclusion of a highly specific AffimerGPC3 capture reagent resulting in sensitivity and specificity parameters for 62.5% and 92.3% in 276 clinical samples, respectively.

Taking IHC results as the “gold standard control”, our proposed assay was satisfactorily consistent with a sensitivity of 81.25% and a specificity of 100%. While there remain 18.75%(6/32) of serum samples that gave a false negative result, this may be due to the absence of GPC3 fragments in serum if the GPC3 protein has not been released from the cell surface into the extracellular environment, as studies have shown previously^7, 34^. In contrast, whilst the sensitivity of the dual-MAbs CLIA kit was a little higher (90.62%) the specificity was very poor (0/9). It seems the combination of an Affimer and monoclonal antibody provided an increase in the specificity of detection over the dual-MAbs CLIA kit from DaRui.

The correlation between serum AFP and GPC3 showed that GPC3 would be positive not only in AFP positive HCC patients, but also in AFP negative HCC patients, and the combination of AFP and GPC3 could increase the sensitivity for HCC diagnosis, which correlates with a previous report^12^.

In conclusion, we have successfully established a new approach for the development of a diagnostic test using a combination of a novel non-antibody binding protein and a traditional monoclonal antibody for detection of serum GPC3. This approach of substituting a non-antibody binding protein in place of traditional antibody in immunoassay development may be of more general value.

## Methods

### Tissue and serum specimens

A total of 179 serum samples were obtained from patients at the First Affiliated Hospital of Zhejiang University between January 2014 and November 2015, including 80 with HCC (ages 32–78 years), 50 with liver cirrhosis (ages 33–67 years), 10 with hepatitis B (ages 26–69 years), 10 with hepatitis C (ages 31–59 years) and 29 with intrahepatic cholangiocarcinoma (ages 30–66 years). Forty-one HCC tissues and paired para-carcinomas were also obtained from the some of the 80 patients with HCC. All of the HCC patients were diagnosed based on histopathological findings. In addition, 196 serum samples and another 325 serum samples were collected from healthy volunteers, aged from 16–48 years old, who had their blood drawn at the DaAn Clinical Laboratory Center of SUN YAT-SEN University between November 2014 and November 2015. All serum specimens were stored at −80 °C before use. Peripheral blood samples and tumor tissues from patients were obtained with informed consent from all subjects, and the research activity was approved by the Ethical Committee (EC) of the First Affiliated Hospital of Zhejiang University (REC number: EC-2015-82). All methods for sample collection and experiments were performed in accordance with the relevant guidelines and regulations as approved by the EC of the First Affiliated Hospital of Zhejiang University.

### Chemicals, immunoreagents and apparatus

Bovine serum albumin (BSA) was acquired from Bovogen Biologicals Pty Ltd (Victoria, Australia). N-hydroxysulfosuccinimide (NHS), 4-morpholineethanesulfonic acid (MES), streptavidin (SA), 1-ethyl-3-(3-dimethylaminopropyl) hydrochloride (EDC), proclin-300 and Tween-20 were obtained from Sigma-Aldrich (St. Louis, MO, USA). Acridinium ester (AE) was purchased from Wallac Company (Turku,Finland). EZ-Link Sulfo-NHS-LC-Biotin was acquired from Thermo Fisher Scientific Inc (Waltham, MA, USA). Magnetic beads were purchased from JSR Life Sciences Corporation (Ibaraki, Japan). Protein markers were purchased from Life Sciences Co.Ltd (St, Petersburg, FL, USA). Chemiluminescent activation fluids (pre-trigger and trigger) were purchased from United Medical Instruments Co. Ltd (Dalian, China). Sephadex G-50 was obtained from Amersham Pharmacia Biotech (Piscataway, NJ, USA). A magnetic separator was obtained from Tianjin Baseline Chromtech Research Centre (Tianjin, China). The automatic chemiluminescence immune-analyzer was purchased from Xiamen UMIC Medical Instrument co. LTD (Xiamen, China). GPC3 recombinant protein and anti-GPC3 monoclonal antibodies (Nos 7D11 and 8G6) were prepared as described in our previous study^13^. The mAb-7D11. mAb-8G6, and Affimer-GPC3-22, were isolated against recombinant GPC3 protein (72–369 aa). GPC3 enzyme-linked immuno-sorbent assay (ELISA) kits were offered by Abnova (Taiwan, China) and R&D Systems (Minneapolis, MN, USA). GPC3 immunohistochemistry (IHC) kit was a kind gift from LBP Medicine Science & Technology Co, Ltd (Guangzhou, China). The traditional dual MAbs-CLIA kit and AFP quantitative diagnostic kit (CLIA) were provided by DaRui Biotechnology Co., Ltd (Guangzhou, China). All solvents were analytical grade. Ultra-pure water was obtained using a Milli-Q water purification system (Millipore, Bedford, MA, USA).

### Solutions

A binding buffer (0.1 mol/L MES pH 5.0) was used to covalently crosslink protein to magnetic beads. The blocking solution contained 50 mM Tris–HCl, 0.9% NaCl, 0.04% proclin-300, 2.5% BSA, and 3% trehalose (pH 7.8). The washing buffer consisted of phosphate-buffered saline (PBS: 0.01 M KH2PO4, 0.01 M Na2HPO4, pH 7.8) containing 0.01% Tween-20. The AE-labeling buffer was 0.2 M phosphate buffer (pH 6.3 and 7.2).The dilution buffer was TBST (pH 7.8) with 0.1 M Tris-HCl, 0.15 M NaCl, 2.5% BSA, and 0.05% Tween-20.

### Phage display and Affimer selection

The GPC3 protein was biotinylated and presented for phage display as previously described^14^. In brief, three panning rounds were performed on streptavidin or neutravidin coated plates and streptavidin coated magnetic beads. After the final panning, 32 monoclonal Affimer reagents were tested for binding against the target protein by phage ELISA. To determine the number of unique monoclonal reagents, all the Affimer clones that showed binding to GPC3 were subjected to DNA sequence analysis. The coding regions for all the unique reagents were then cloned into the protein expression vector pET11a, recombinant protein was produced in BL21 (DE3) cells and purified using nickel NTA resin. The Affimer coding regions were cloned into pET11, and Affimer protein produced and purified as previously described^14^.

### Preparation of coated magnetic beads with Affimer

Magnetic beads were coated using a method described in a protocol provided by the manufacturer (JSR, Japan). First, 8 mg magnetic beads were placed into a micro tube and washed five times with 200 μL of binding buffer. In order to activate carboxyl groups on the magnetic bead surfaces, 25 μL fresh EDC of 10 mg/ml and 40 μL NHS of 10 mg/mL were added to magnetic beads, which were then incubated with vortex mixing at room temperature for 30 min. After that, the supernatant was discarded and the beads were washed three times with 200 μL of binding buffer. Next, 0.1 mg of GPC3-22 and 8 mg of activated magnetic beads were added in 1 mL of binding buffer and mixed by vortexing overnight at room temperature. The following day, the magnetic bead/GPC3-22 conjugates were blocked with 1 mL of blocking buffer, after which they were washed three times with blocking buffer and stored in the same buffer at 4 °C until use.

### Biotinylation of antibody

Antibody (0.5 mg) was dissolved with 0.2 mol/L phosphate buffered (pH 7.2) and the precipitate was collected by centrifugation (9000 rpm * 6 min). 50 μL NHS-LC-Biotin solution (1 mg/mL) was then added. The reaction mixture was incubated with shaking for 2 h at room temperature. Then the labeled antigen mixture was dialyzed in 0.2 mol/L phosphate buffer (pH 7.2) overnight. After adding 5% Proclin-300 as a protectant, aliquots of conjugates were diluted to a volume proportion of 1:250 with TBST (pH 7.8) and stored at 4 °C for daily use.

### Preparation of coated AE with SA

Streptavidin (0.5 mg) was dissolved with 200 μL 0.2 mol/L phosphate buffered (pH 6.3) and the precipitate was collected by centrifugation at 8,000 rpm for 5 min for five times. Then the concentration was adjusted to 1 mg/ml with the same buffer. 1 μL AE solution (10 mg/mL) was added and mixed well, the reaction mixture was performed in the dark for 12 h at room temperature. The labeled SA mixture was purified by Sephadex G-50 using 0.2 mol/L phosphate buffer (pH 6.3). Finally, 5% Proclin-300 solution was added to the purified SA solution. The purified labeled SA were diluted to a volume ratio of 1:500 with TBST (pH 7.8) and stored at 4 °C.

### Assay procedure

The assay is a sandwich CLIA that uses Affimer coated magnetic beads to capture GPC3 antigens, acridinium ester (AE)-labeled streptavidin (SA) and biotinylated anti-GPC3 antibodies as detection reagents. The assay comprised 60 μL of magnetic beads coated with Affimers, 60 μL of a serum sample or standard, 100 μL of biotinylated anti-GPC3 antibody dispensed into the reactive wells in a stepwise manner, and then incubated for 15 min at room temperature. Each well was washed twice with washing buffer, and 100 μL of AE-labeled SA was added. After incubation at room temperature for 10 min, each well was washed twice and filled with pre-trigger solution containing H_2_O_2_ (pH < 2). The chemiluminescence value was measured when the trigger solution (NaOH solution, pH > 13.0) was added to each well.

### Optimization of reaction parameters

In order to determine optimal reaction parameters for the immunoassay, various factors including pH of the dilution buffer, the concentration of GPC3-22 coated magnetic beads, the ratio of 7D11 and NHS-LC-Biotin, the amount of AE-labeled SA, and the volume of serum sample/standard were optimized. The optimum reaction parameters were obtained by the checkerboard titration experiments^35^.

### Analytical performance assessment

The typical parameters for analytical performance were assessed, including detection limitation, hook effect, accuracy, specificity, precision and stability. The assessment methods were as described in our previous study^10^.

### Clinical sample detection

Serum samples were analysed by the proposed assay, and by dual-MAbs ELISA kits (GPC3(Human) ELISA kit, Cat# KA 1175, from Abnova; Quantikine® ELISA Human Glypican 3 Immunoassay, Cat# DGLY30, from R&D systems Inc.) and the dual-MAbs CLIA kit (Glypican-3 (GPC3) quantitative diagnostic kit (CLIA), and AFP quantitative diagnostic kit (CLIA), from DaRui Biotechnology Co., LTD, Guangzhou, China). Tissue samples were detected using the IHC kit from LBP Medicine Science and Technology Co. LTD. (GuangZhou China). The assay protocols are all provided by the manufacturer’s product instructions.

### Statistics

SPSS 21 (Chicago, USA) and Origin Pro7.5 (Microcal, USA) were used to analyze the data. Area under the curve (AUC) analysis was performed for determining the diagnostic value for HCC. The diagnostic cut-off values were determined by the percentile method, the receiver-operating curve (ROC), as well as from the literature. Linear regression and Spearman’s correlation were performed to analyze the results of quantitative data between two methods, and for Kappa value results of qualitative data. Significant difference was set at P < 0.05.

### Data Availability

All data generated or analysed during this study are included in this published article and the supplementary information files.

## Electronic supplementary material

Supplementary Figures and Tables
