# Supplementary material for: Development of an Affimer-antibody combined immunological diagnosis kit for glypican-3
Source: Sci Rep. 2017 Aug 29;7:9608. doi: 10.1038/s41598-017-10083-w (PMC5575301; doi:10.1038/s41598-017-10083-w)
Supplement: Supplementary file 1 — Supplementary Figures and Tables [file 41598_2017_10083_MOESM1_ESM.pdf]

Development of an Affimer-antibody combined immunological diagnosis kit for glypican-3

Chunmei Xie[1,+], Christian Tiede [2,+], Xuanyi Zhang[4], Congrong Wang[5], Zhixiong Li[6], Xiao Xu[4], Michael J McPherson[2,3] , Darren C.Tomlinson [2,3*] ,Weiwen Xu[1*]

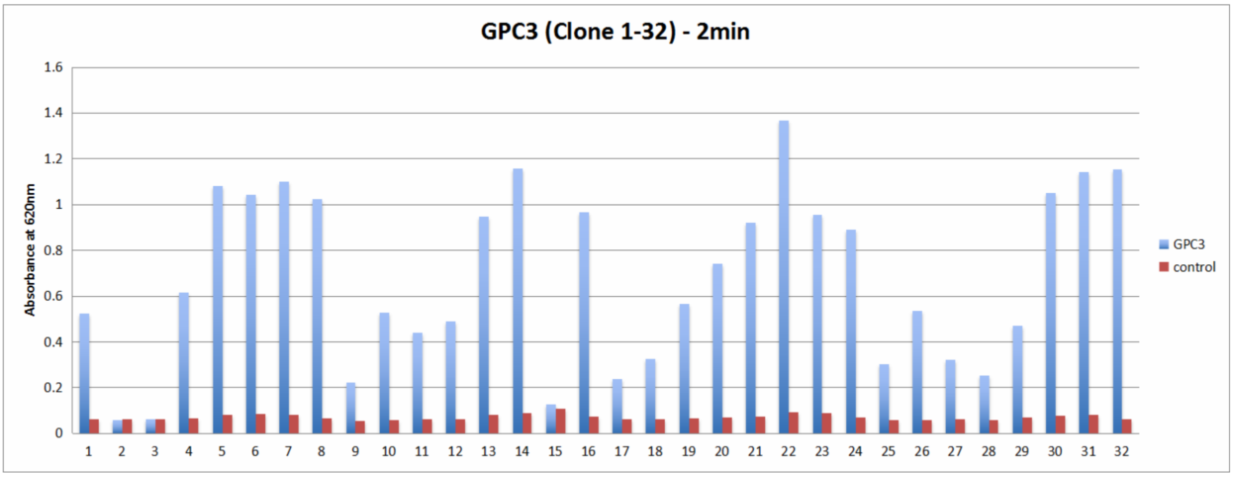

Supplementary Fig. S1. Results of bio-panning from phage-display library with GPC3 by phage-ELISA.

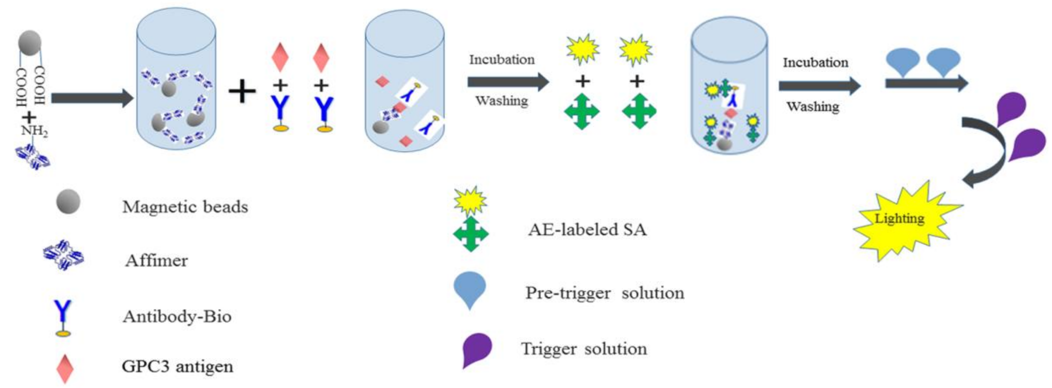

Supplementary Fig. S2. Schematic representation of the Affimer-MAb CLIA for detecting GPC3.

Supplementary Table S1. Screening results of paired reagents with SNR values.

L R<br>C R	GPC3-1	GPC3-4	GPC3-6	GPC3-21	GPC3-22	GPC3-25	8G6	7D11
GPC3-1	-	24.34	34.70	45.00	69.00	44.17	30.90	123.45
GPC3-4	98.02	-	34.20	65.78	65.34	34.20	29.78	450.66
GPC3-6	56.67	78.98	-	87.67	45.36	67.12	33.46	342.10
GPC3-21	17.24	20.77	24.24	-	50.68	33.45	28.00	30.28
GPC3-22	965.58	989.46	900.88	340.78	-	567.45	358.98	<u>1320.55</u>
GPC3-25	418.66	337.81	257.01	33.42	78.50	-	64.45	247.46
8G6	78.96	45.34	650.00	120.00	78.90	55.60	-	356.98
7D11	45,65	78.91	98.01	78.09	66.78	67.12	780.91	-

LR: Labeling reagents , CR: Coating reagents. GPC3-1,GPC3-4, GPC3-6,GPC3-21,GPC3-22 and GPC3-25 represent Affimers.8G6 and 7D11 are monoclonal antibodies.

Supplementary Table S2. Recovery rates of the proposed CLIA assay(n=3).

Original concentration of GPC3（ng/mL）	Added concentration of GPC3（ng/mL）	Measured concentration of GPC3（Mean±SD ng/mL）	Recovery（%）
0.5	50	46.40±2.23	91.88
	300	286.80±18.34	95.44
1	50	48.37±3.09	94.84
	300	305.80±16.08	101.59
2	50	49.85±2.90	95.87
	300	315.60±19.81	104.50

Supplementary Table S3. Serum levels of GPC3 and AFP in different groups.

Group	N	AFP (Mean±SD ng/mL)	GPC3 (Mean±SD ng/mL)
NP	325	75.35±922.15	0.23±0.62
HCC	80	6105.64±18271.30	7.30±11.42
ICC	29	10.70±20.99	0.07±0.19
HB/HC	20	10.84±29.17	0.23±0.38
LC	50	1324.62±7535.15	0.28±0.57

NP: normal population. HCC: hepatocellular carcinoma. ICC: intrahepatic cholangiocarcinoma. HB: hepatitis B. HC: hepatitis C. LC: liver cirrhosis.

Supplementary Table S4. Comparison of diagnostic values about individual and combined tests of AFP and GPC3 for diagnosis of HCC

Item combination	Sensitivity（%）	Specificity（%）	Accuracy rate (%)
AFP	57.50(46/80)	88.92(377/424)	83.93(423/504)
GPC3	62.50(50/80)	93.40(396/424)	88.49(446/504)
AFP+GPC3	87.50(70/80)	82.55(350/424)	83.33(420/504)
